# Antiretroviral therapy and associated drug interactions with cardiovascular drugs: a critical review

**DOI:** 10.3389/fphar.2025.1685710

**Published:** 2025-11-21

**Authors:** Marios Spanakis, Konstantinos Alexakis, Petros Ioannou

**Affiliations:** 1 Division of Social Medicine, School of Medicine, University of Crete, Heraklion, Greece; 2 Computational Bio-Medicine Laboratory, Institute of Computer Science, Foundation for Research and Technology – Hellas, Heraklion, Greece; 3 German Medical Institute, Limassol, Cyprus; 4 Department of Internal Medicine and Infectious Diseases, University Hospital of Heraklion, Heraklion, Greece; 5 School of Medicine, University of Crete, Heraklion, Greece

**Keywords:** HIV, AIDS, drug–drug interactions, people living with HIV, antiretroviral treatment, cardiovascular disease, Lexidrug^®^, Liverpool Drug Interaction Checker

## Abstract

The significant progress in HIV research has led to the development of innovative medicines and protocols. Most people living with HIV (PLWH) achieve longer lifespans when adhering strictly to the therapeutic protocols. A notable characteristic of PLWH is a higher incidence of cardiovascular diseases. This may result from the disease itself or increased life expectancy. Regardless of the underlying cause, it often requires the concurrent use of antiretroviral therapy (ART) and cardiovascular disease (CVD) medications. In such cases, a key issue regarding optimal evidence-based practices is the management of potential and clinically significant drug–drug interactions (DDIs). Over time, our understanding of DDIs has advanced significantly. These advancements have led to the development of tools and approaches for identifying and managing DDIs, especially in special population groups such as PLWH. Clinically, recognizing and managing these interactions is key to preventing adverse effects and optimizing outcomes in PLWH, thereby enhancing patient safety and quality of care. This review discusses the latest data on DDIs between ART and CVD medications, emphasizing their clinical significance. Furthermore, it explores how established tools, such as the Liverpool Drug Interaction Checker and Lexidrug®, can support healthcare professionals. Combined with insights from the literature and summaries of product characteristics, these tools can guide the identification and management of DDIs between ART and CVD medications to ensure optimal therapeutic outcomes.

## 1 Introduction

People living with HIV (PLWH) numbered approximately 40 million in 2023, according to the Joint United Nations Program on HIV/AIDS (UNAIDS), with 30.7 million receiving antiretroviral therapy (ART) (Fact sheet—Latest global and regional statistics on the status of the AIDS epidemic, 2024). Since its declaration as a pandemic, extensive research has led to the development of innovative antiretrovirals, significantly improving patient outcomes (Gandhi et al., 2023; Tseng et al., 2015). The success of ART regimens has led to an aging population of PLWH, who are also at increased risk for age-related diseases and other non-HIV-related comorbidities, necessitating evidence-based healthcare approaches (Nicolau et al., 2023).

Cardiovascular disease (CVD) is a significant concern in this population as it is recognized as a leading cause of non-HIV-related morbidity and mortality (Feinstein et al., 2016). PLWH are at a higher risk of CVD due to the combined effects of disease progression, treatment side effects, and extended life-expectancy. Epidemiological studies have shown that PLWH have a 1.5–2-fold increased risk of myocardial infarction compared to uninfected individuals in Europe and the United States (Shah et al., 2018; Triant, 2013). This necessitates careful management of both HIV and CVD to optimize patient outcomes, as reflected, for example, in guidelines for CVD risk reduction via lipid modulation treatment (Mach et al., 2020). The findings from the REPRIEVE trial showed that pitavastatin significantly lowered the risk of major adverse cardiovascular events in PLWH with low-to-moderate CVD risk over a median of 5 years (Grinspoon et al., 2023).

The growing need for CVD treatment in PLWH raises concerns about polypharmacy. It also highlights the potential for drug–drug interactions (DDIs) between ART and other medications, which may lead to adverse drug reactions (ADRs) (Edwards and Aronson, 2000; Marin et al., 2023; Peng et al., 2024). DDIs refer to alterations in drug exposure or response due to the co-administration of another drug (McQuade and Campbell, 2021). The clinical relevance of DDIs refers to the risk for ADRs or alterations in therapeutic efficacy (Scheife et al., 2015). DDIs occur through pharmacokinetic (PK-DDIs) mechanisms such as changes in absorption, distribution, metabolism, and elimination (ADME) processes or through pharmacodynamic (PD-DDIs) mechanisms involving additive, synergistic, or antagonistic effects of interacting drugs (Hines et al., 2012; Niu et al., 2019). Key PK-DDI mechanisms involve transporters such as P-glycoprotein (P-gp) and organic anion-transporting polypeptides (OATPs) and enzymes such as cytochrome P450 (CYP) and UDP-glucuronosyltransferases (UGTs) (Palleria et al., 2013; Yu et al., 2019). P-gp is a membrane transporter found in the intestine, liver, and other tissues. It plays a crucial role in drug absorption and elimination. Changes in P-gp activity can affect absorption and overall drug bioavailability. In HIV infection, transporters such as P-gp play a more dynamic role. Studies show altered protein density and gene expression in the gastrointestinal tract during treatment. ART-treated patients often exhibit increased P-gp expression, which may affect the DDI dynamics (De Rosa et al., 2013; Kis et al., 2016). Regarding drug metabolism, CYP enzymes, especially CYP1A2, CYP2C9, CYP2C19, CYP2D6, and CYP3A4/5, are responsible for nearly 80% of phase-I metabolism and over 50% of drug elimination (excluding biologics), with CYP3A4/5 alone metabolizing 40%–50% of all medications (Teo et al., 2015; Zanger and Schwab, 2013).

DDIs involving CYPs are widely studied, as changes in metabolism can significantly alter drug concentrations (Loos et al., 2022; Naccarato et al., 2014). Similarly, UGTs facilitate drug or phase-I metabolite elimination *via* glucuronidation (phase-II metabolism), and DDIs with UGTs can also modulate drug concentrations, leading to changes in the PK profiles (Jancova et al., 2010; McCance-Katz et al., 2010).

This review examines the current literature on potential DDIs between ART drugs and cardiovascular medications. It also incorporates data from drug interaction tools often used in clinical practice to identify and manage these interactions (Abarca et al., 2006; Roblek et al., 2015). Drug interaction checkers, such as the evidence-based “HIV Drug Interaction Checker” (University of Liverpool) and the subscription-based Lexidrug® database, are key resources that provide real-time information on the interaction potential and clinical significance (University of Liverpool, 2024; Uptodate Lexi-drug, 2024). In this review, both tools were used to identify potential DDIs between ART and CVD medications comprehensively. This dual approach aimed to enhance sensitivity, validate identified DDIs, and ensure comprehensive data coverage. Moreover, it allows us to identify any potential discrepancies that we aim to resolve, facilitating an advanced analysis of the interplay between ART drugs and potentially interacting CVD drugs. By integrating these tools with a detailed literature review, this study identifies, evaluates, and synthesizes relevant data. It also addresses potential discrepancies to inform clinical practice and guide the co-administration of ART and CVD medications.

## 2 Methods

The ART and CVD medications analyzed in this study are presented in Table 1. DDIs were assessed using the “HIV Drug Interaction Checker” developed by the University of Liverpool (UoL) and Lexidrug® (formerly Lexicomp®) software. Interactions were categorized as PK-DDIs or PD-DDIs. The UoL database categorizes the significance of potential DDIs into four categories as follows: “no interaction,” “potential weak interaction,” “potential interaction,” and “do not administer.” Lexidrug® categorizes the significance of potential DDIs as “no interaction, A”; “no action needed, B”; “potential weak interaction—monitor therapy, C”; “potential interaction—consider modifications, D”; and “do not administer, X.” For consistency, Lexidrug®’s A and B categories were grouped as “no interaction,” thus aligning both tools in four unified categories.

**TABLE 1:** Drug categories and compounds assessed for potential DDIs.

ART category	Compound	CVD category	Compound
Protease inhibitors	Amprenavir Atazanavir Darunavir Fosamprenavir Indinavir Lopinavir Nelfinavir Ritonavir Saquinavir Tipranavir	ACE inhibitors	Benazepril Captopril Enalapril Fosinopril Lisinopril Perindopril Quinapril Ramipril Trandolapril
Nucleoside and nucleotide reverse-transcriptase inhibitors (NRTIs)	Abacavir Didanosine Emtricitabine Lamivudine Stavudine Tenofovir alafenamide Tenofovir disoproxil Zidovudine	Angiotensin II receptor blockers (ARBs)	Azilsartan Candesartan Eprosartan Irbesartan Losartan Olmesartan Telmisartan Valsartan
Non-nucleoside reverse-transcriptase inhibitors (NNRTIs)	Doravirine Efavirenz Etravirine Nevirapine Rilpivirine	Antiarrhythmics	Amiodarone Bepridil Digoxin Disopyramide Dronedarone Flecainide Lidocaine Mexiletine Propafenone Quinidine
Integrase inhibitors	Cabotegravir Dolutegravir Elvitegravir Raltegravir	Anticoagulants	Acenocoumarol Apixaban Dabigatran Edoxaban Rivaroxaban Warfarin
Integrase strand-transfer inhibitor (INSTI)	Bictegravir	Anti-platelet	Aspirin Clopidogrel Dipyridamole Prasugrel Ticagrelor Ticlopidine
HIV entry inhibitor	Fostemsavir	Calcium channel blockers (CCBs)	Amlodipine Felodipine Lacidipine Nifedipine Verapamil Diltiazem
HIV fusion inhibitor	Enfuvirtide	Diuretics	Amiloride Furosemide Spironolactone Eplerenone
Assembly inhibitor	Lenacapavir	HMG CoA reductase inhibitors	Atorvastatin Fluvastatin Lovastatin Pitavastatin Pravastatin Rosuvastatin Simvastatin
CCR5 receptor antagonist	Maraviroc	β-blockers	Atenolol Bisoprolol Carvedilol Metoprolol Nebivolol Oxprenolol Pindolol Propranolol Sotalol
Other	Cobicistat		

Abbreviations: ACE, angiotensin converting enzyme; ART, antiretroviral; CCR5, C–C chemokine receptor type 5; CVD, cardiovascular; DDI, drug–drug interaction; HIV, human immunodeficiency virus.

After the software-based evaluation, a comprehensive literature review was conducted. The sources included MEDLINE, regulatory reports, and summaries of product characteristics (SmPCs). The MEDLINE search utilized medical subject heading (MeSH) terms and keywords such as ‘HIV,’ ‘antiretroviral therapy,’ ‘protease inhibitors,’ ‘statins,’ ‘beta-blockers,’ ‘angiotensin II receptor blockers (ARBs),’ ‘angiotensin converting enzyme (ACE) inhibitors,’ ‘antihypertensives,’ ‘cardiovascular drugs,’ and ‘drug–drug interactions,’ focusing on PK/PD mechanisms, clinical outcomes, and risk for adverse effects. The review covers studies up to May 2024. It synthesizes evidence on CVD risk in PLWH, the assessment and management of DDIs between ART and CVD drugs, and the discrepancies in interaction information.

## 3 Results

### 3.1 Interactions identified by DDI checkers

**FIGURE 1:**
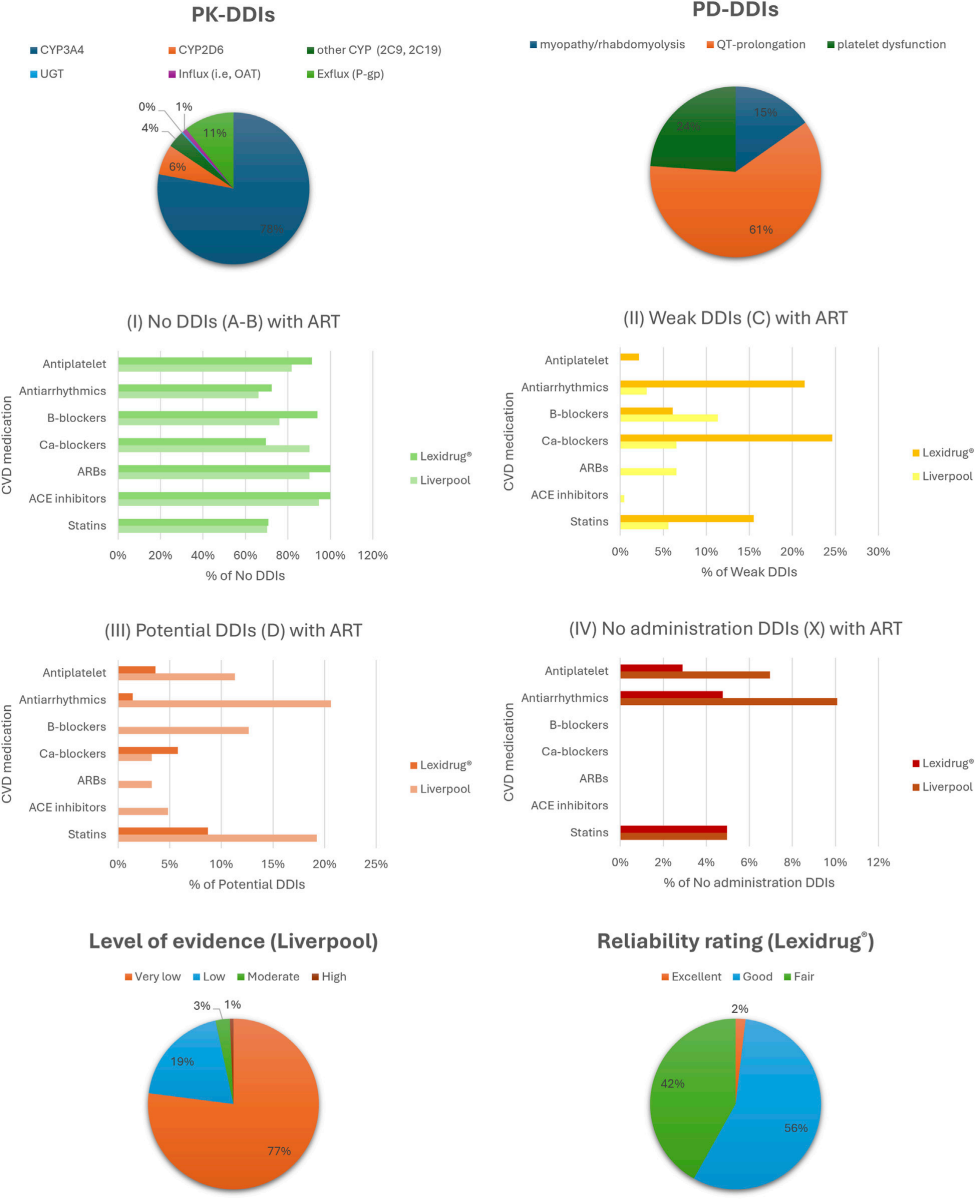
Analysis of DDIs using the two checkers, focusing on pharmacokinetic (PK) and pharmacodynamic (PD) mechanisms, clinical significance, and the level of evidence supporting the data. Abbreviations, ARBs: angiotensin II receptor blockers; ACE, angiotensin converting enzyme; Ca-blockers, calcium channel blockers; CYP, cytochrome P450; OAT, organic anion transporter; UGT, UDP-glucuronosyltransferase; P-gp, P-glycoprotein.

The UoL database showed 313 potential DDIs, while Lexidrug® identified 365 DDIs. Lexidrug® includes older ARTs such as amprenavir, didanosine, fosamprenavir, nelfinavir, nevirapine, saquinavir, stavudine, and tipranavir, which are rarely used today. Both checkers attributed 87%–88% of DDIs to PK mechanisms. These mainly involve CYP3A4-mediated metabolism and P-gp-mediated transport. Regarding PD-DDIs, drug-induced arrhythmias due to QT-prolongation, potential clotting disturbances, and the risk of rhabdomyolysis were the most dangerous events. Drugs from both drug classes can act as perpetrators or victim drugs depending on the increased exposure or prolonged action. Differences in how each checker reports the clinical significance of DDIs are also noted (Figure 1). All the identified potential DDIs are presented and discussed in the following sections. Table 2 provides a summary of those requiring avoidance, monitoring, or dose adjustment, as determined in the current analysis.

**TABLE 2:** Clinically significant DDIs require avoidance, monitoring, or dose adjustment as determined in the current work. “Avoid” indicates that co-administration is contraindicated. “Monitor” indicates that clinical or laboratory monitoring is recommended. “Adjust dose” indicates that dose modification is necessary to reduce risk. Details are discussed in the related sections.

ART class	CVD	Action/Recommendation	Pharmacological mechanisms
NRTIs	Tenofovir + amiodarone	Monitor renal function	P-gp inhibition may ↑ tenofovir exposure
	Tenofovir + verapamil	Monitor/adjust dose	Risk ↑ nephrotoxicity
NNRTIs	Efavirenz + amiodarone	Avoid/monitor ECG	CYP3A4 induction → altered antiarrhythmic levels
	Efavirenz + rivaroxaban	Avoid/monitor	↑ Bleeding risk via CYP3A4/P-gp induction
INSTIs	Dolutegravir + atenolol	Monitor BP	OCT2/MATE1 inhibition → ↑ atenolol exposure
	Bictegravir + disopyramide	Monitor ECG	Low but possible toxicity
PIs (boosted)	Ritonavir + simvastatin/Lovastatin	Avoid	↑ Rhabdomyolysis risk via CYP3A4 inhibition
	Ritonavir + amiodarone	Avoid/monitor ECG	CYP3A4 inhibition → QT prolongation
	Ritonavir + rivaroxaban	Avoid	↑ Bleeding risk
	Ritonavir + eplerenone	Avoid	Severe hyperkalemia risk
Attachment–post attachment inhibitors and entry inhibitors	Maraviroc + verapamil/Diltiazem	Adjust/monitor BP	↑ Plasma levels via CYP3A4 inhibition
	Fostemsavir + statins (except pravastatin)	Monitor/adjust dose	↑ Statin exposure via OATP/BCRP inhibition

### 3.2 DDIs by ART category

#### 3.2.1 Nucleoside reverse transcriptase inhibitors

**FIGURE 2:**
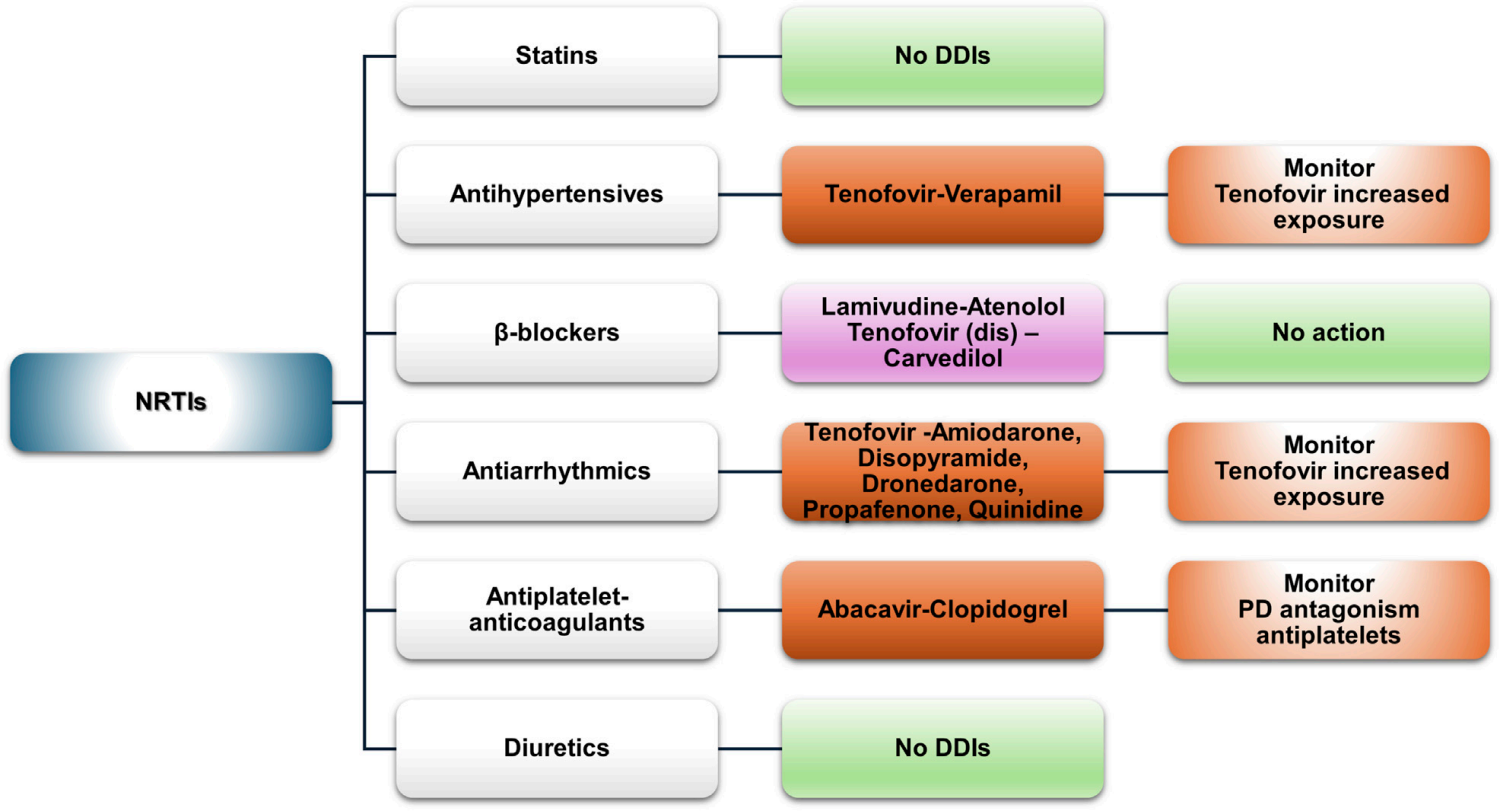
An overview of the potential DDIs between NRTIs and CVD medications with the most prominent cases and the suggested action according to the checkers used and literature data (green, no DDIs; light purple, potential but of low significance; red, clinically significant).

Nucleoside reverse transcriptase inhibitors (NRTIs) are considered essential components in the management of HIV infection (EACS Guidelines | EACSociety, 2024; HIV/AIDS Treatment Guidelines|Clinicalinfo.HIV.gov, 2024). NRTIs undergo intracellular phosphorylation, which converts them into active metabolites capable of inhibiting HIV’s reverse transcriptase (Holec et al., 2017). NRTIs are substrates of multidrug and toxin extrusion (MATE) and organic cation transporters (OCTs). These transporters influence their cellular uptake and elimination. Emtricitabine and lamivudine are well-known examples of NRTIs that utilize the MATE or OCT transporters for their intracellular transport (Cano-Soldado et al., 2004; Jung et al., 2008; Reznicek et al., 2017; Truong et al., 2008). This mechanism contributes to the efficacy and safety of NRTIs. Their transport helps maintain adequate intracellular drug levels while minimizing systemic toxicity. Generally, NRTIs show limited interaction potential (Figure 2).

##### 3.2.1.1 Statins

No DDIs with statins are expected since NRTIs are not substrates or inhibitors of CYP3A4 or OATP1B1, enzymes known to be implicated in DDIs with statins (VIREAD ACCESS—tenofovir disoproxil fumarate (TDF) tablet, coated, 2021). However, NRTIs are often co-administered with other antiretrovirals that may interact with statins.

##### 3.2.1.2 Antihypertensives (ACE inhibitors, ARBs, and CCBs)

NRTIs have a favorable, low-interaction profile. An exception is verapamil, which inhibits P-gp, potentially increasing tenofovir exposure and the risk of nephrotoxicity (VIREAD ACCESS—tenofovir disoproxil fumarate tablet, coated, 2021). Database comparison revealed minor discrepancies. The UoL database classified the DDI as “amber,” suggesting possible dose modifications, while Lexidrug® listed it as “potentially weak.” The prescribing information for tenofovir alafenamide indicates that it is a substrate of P-gp and breast cancer resistance protein (BCRP) (DESCOVY—emtricitabine and tenofovir alafenamide tablet, 2022). Although the risk is low, caution is advised when co-administering P-gp inhibitors such as verapamil. Increased monitoring or a lower initial dose of tenofovir alafenamide (for example, 10 mg instead of 25 mg) could be considered a precautionary measure as it is suggested for other similar cases (Huliciak et al., 2023).

##### 3.2.1.3 β-blockers

The UoL checker identified a “potential weak” DDI between lamivudine and atenolol. This may result from competition between lamivudine and renal transport proteins (OCT2 and MATE1). However, according to the prescribing information, lamivudine at therapeutic concentrations is not expected to affect the pharmacokinetics of drugs that are substrates of OATP1B1/3, BCRP, P-gp, MATE1, MATE2, OCT1, OCT2, or OCT3 (EPIVIR—lamivudine tablet, film-coated, 2020). Lexidrug® reports a DDI between carvedilol and tenofovir disoproxil due to P-gp inhibition (Lymperopoulos et al., 2015). However, carvedilol is primarily glucoronidated by UGTs (1A1, 2B4, and 2B7) (CARVEDILOL tablet, film-coated, 2024). Therefore, a significant DDI with tenofovir is unlikely. These DDIs, as described in the SmPCs, are likely non-significant. No action is required unless signs of toxicity appear.

##### 3.2.1.4 Antiarrhythmics (CCBs)

P-gp inhibition drives the potential DDIs observed in our analysis, with tenofovir as the “victim” drug. Amiodarone, disopyramide, dronedarone, propafenone, and quinidine were identified as potential DDI perpetrators. The UoL database classifies these as “amber,” suggesting using the 200/10 mg formulation of F/TAF when co-administered with amiodarone or quinidine (Huliciak et al., 2023). Lexidrug® does not provide data for that combination. However, for TDF, it classifies the DDI as potentially weak (yellow), which differs from the UoL database (amber DDI). Similar information is provided for dronedarone and propafenone (Mendell et al., 2013; MULTAQ-dronedarone tablet, film-coated, 2023; PROPAFENONE HCL-propafenone hydrochloride tablet, film-coated, 2019). Monitoring for nephrotoxicity (Fanconi syndrome being one of the manifestations) is advised. No data are available on bone density and on whether it can be significantly affected by prolonged exposure to increased tenofovir concentrations due to such an interaction.

##### 3.2.1.5 Antiplatelets and anticoagulants

Antiplatelet medications such as ASA, clopidogrel, dipyridamole, prasugrel, ticagrelor, and ticlopidine show no DDIs with NRTIs. A potential PD-DDI exists between abacavir and clopidogrel. Abacavir activates platelets, potentially reducing clopidogrel’s effect, although this has been observed only *in vitro* (WHOPAR Part 4 November 2022 On ABACAVIR, n. d.). This observation refers to the abacavir-alone formulation (HA674) and is not found in the SmPCs of Ziagen® or other combination products (ZIAGEN—abacavir sulfate tablet, film-coated, 2023). No DDIs are expected between NRTIs and vitamin K inhibitors (acenocoumarol and warfarin) or direct oral anticoagulants (DOACs) (apixaban, edoxaban, dabigatran, and rivaroxaban).

##### 3.2.1.6 Diuretics

No DDIs were found between NRTIs and loop or potassium-sparing diuretics, thiazides, and mineralocorticoid receptor antagonists (MRAs).

#### 3.2.2 Non-nucleoside reverse transcriptase inhibitors

**FIGURE 3:**
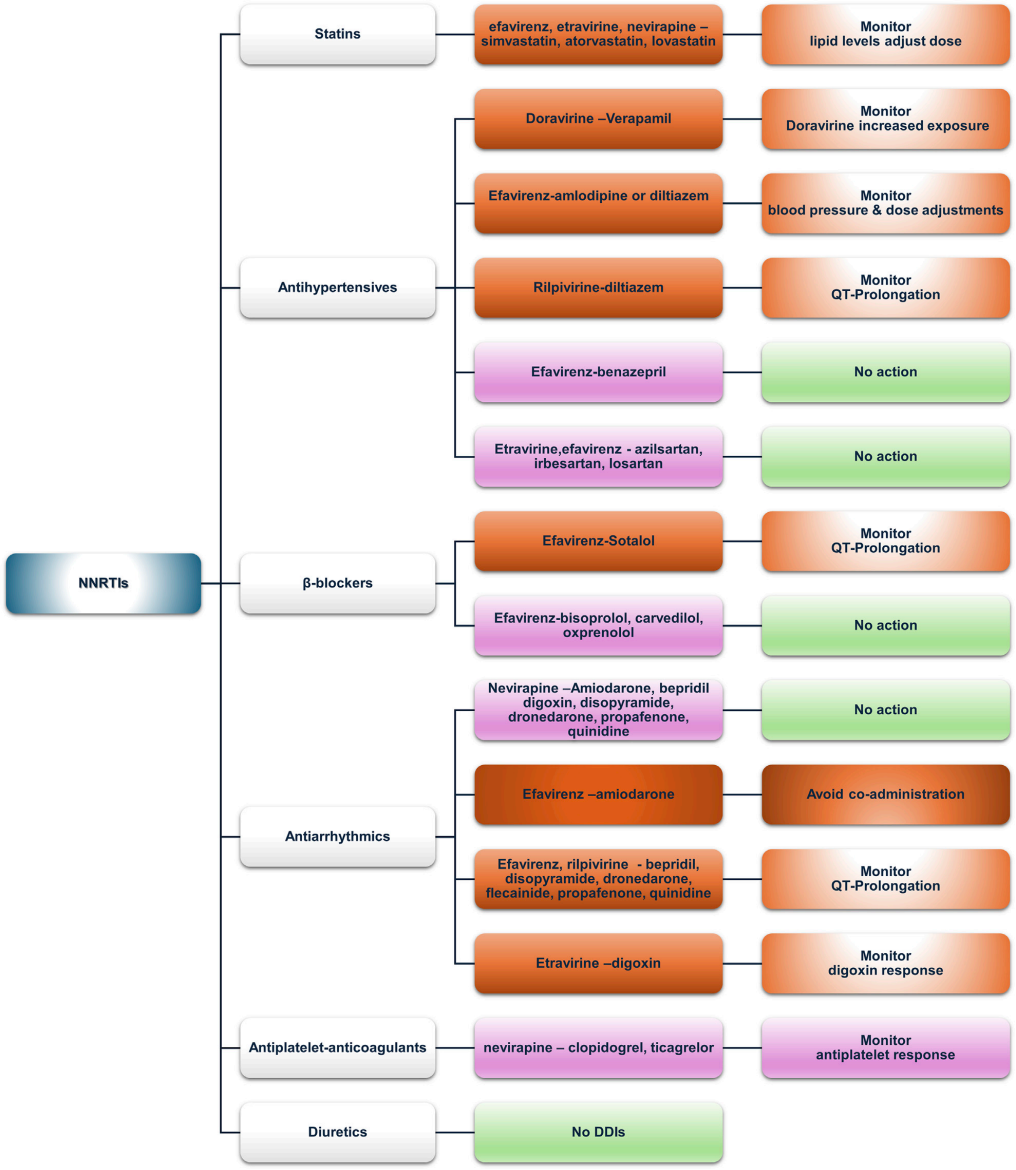
An overview of the potential DDIs between NNRTIs and CVD medications with the most prominent cases and the suggested action according to the checkers used and literature data (green, no DDIs; light purple, potential but of low significance; red, clinically significant; dark red, avoid).

Non-nucleoside reverse transcriptase inhibitors (NNRTIs) are a diverse group of medicines that incapacitate HIV reverse transcriptase and are primarily metabolized *via* the CYP enzymes (Riska et al., 1999). Most of the potential DDIs identified by our analysis are due to mechanisms involving CYP-mediated metabolism. As for the DDI potential, doravirine exhibits the most favorable profile of low DDI risk, followed by rilpivirine, with most being PD in effect. Figure 3 summarizes the data presented below.

##### 3.2.2.1 Statins

Doravirine and rilpivirine do not exhibit DDIs with statins, making them an ideal switch for NNRTI-based regimens. Efavirenz, etravirine, and nevirapine may interact with simvastatin and lovastatin and, to a lesser degree with atorvastatin, potentially *via* CYP3A4 induction by efavirenz, thus increasing intrinsic clearance and reducing total exposure of the aforementioned statins (EFAVIRENZ capsule, 2023; Gerber et al., 2005). However, no recommendations are acknowledged in the prescribing information of any of the above-mentioned cases, except for atorvastatin (LIPITOR—atorvastatin calcium tablet, film-coated, 2024a). In patients not achieving their lipid targets, a switch to another NNRTI, such as etravirine (Cano-Soldado et al., 2004; Ciaffi et al., 2015) or doravirine (Johnson et al., 2019; Orkin et al., 2024), could be considered. As for etravirine, although both databases label the interaction with atorvastatin as “yellow,” the SmPC states that co-administration needs no dose adjustments (INTELENCE—etravirine tablet, 2023), which also contrasts with a publication by Kakuda et al. (2011). Similarly, the UoL database reports DDIs for pravastatin, which contrasts with its SmPC.

Rosuvastatin shows low DDI potential due to limited CYP metabolism (INTELENCE—etravirine tablet, 2023). Simvastatin and lovastatin could be safely administered to PLWH to treat dyslipidemia, albeit with dose adjustments (Rahman et al., 2008). Nevirapine, metabolized by CYP3A and CYP2B6, can inhibit CYP3A according to *in vitro* data (Erickson et al., 1999; Ernest et al., 2005; Granfors et al., 2006; Wen et al., 2009) and has an “amber”-class potential of DDIs with simvastatin and lovastatin according to the UoL database but not according to Lexidrug®. However, no data exist in the SmPCs regarding either the above-mentioned statins or nevirapine (LOVASTATIN tablet, 2020; VIRAMUNE-nevirapine tablet, 2022; ZOCOR-simvastatin tablet, film-coated, 2023). In addition, the literature also presents a paucity of relevant data addressing these DDIs, except for a possible case of statin-induced myopathy (Siriangkhawut et al., 2017).

##### 3.2.2.2 Antihypertensives (ACE inhibitors, ARBs, and CCBs)

Doravirine and rilpivirine are victim drugs in terms of DDIs with antihypertensives. The UoL database states that non-dihydropyridine calcium channel blockers (CCBs), such as diltiazem and verapamil, being moderate CYP3A4 inhibitors, can mediate a concentration increase of doravirine or rilpivirine, potentially raising the risk of QTc prolongation (EDURANT-rilpivirine hydrochloride tablet, film-coated,” 2024b). Amlodipine, a CYP3A4 substrate, interacts with inducers (e.g., efavirenz) or inhibitors (e.g., ritonavir-boosted regimens), requiring careful monitoring of blood pressure and dose adjustment—doubling with inducers and halving with inhibitors—as reported in a recent population PK analysis (Courlet et al., 2021). Moreover, co-administration may increase the risk for arrhythmias. Efavirenz and etravirine, as CYP inducers, can reduce CCB exposure, with efavirenz prescribing information stating that dose adjustment should be guided by clinical response for diltiazem and, by inference, other CCBs (EFAVIRENZ capsule, 2023). Even though etravirine and nevirapine do not contain implicit warnings for CCBs, monitoring and adjustments should be made accordingly where no alternative exists.

ACE inhibitors exhibit a favorable profile, which is convenient given their place in the current guidelines for hypertension and heart failure (2023 ESH Guidelines for the Management of Arterial Hypertension, Task Force for the Management of Arterial Hypertension of the European Society of Hypertension, 2024; McDonagh et al., 2021). An exception may be benazepril, a substrate of UGTs, for which limited evidence suggests induction by efavirenz (Lee et al., 2012). The clinical response should guide any decision regarding adjustments.

ARBs also have a generally neutral DDI profile. The UoL database reports potential weak interaction between etravirine or efavirenz and azilsartan, irbesartan, or losartan due to CYP2C9 inhibition that may potentially increase ARB concentrations. However, no dose adjustment is proposed.

##### 3.2.2.3 β-blockers

Doravirine and rilpivirine exhibit no DDI potential with β-blockers in our analysis. Lexidrug® reports no DDIs with other NNRTIs, except a potential weak PD-DDI between efavirenz and sotalol regarding QTc prolongation, as efavirenz can prolong QT *via* inhibition of the hERG current in CYP2B6*6*6 allele carriers (Abdelhady et al., 2016; Castillo et al., 2002; Hosseini et al., 2021) and sotalol has the potential of QTc prolongation as well (Lenhoff et al., 2016). The UoL database identifies an amber-class DDI between efavirenz and etravirine with oxprenolol, potentially due to UGT induction reducing oxprenolol levels, but the evidence is only theoretical (Russo and Covinsky, 1983). Similarly, carvedilol and bisoprolol levels may be affected. Carvedilol levels may either increase *via* slight inhibition of CYP2C9 by etravirine or efavirenz or decrease *via* UGT induction (CARVEDILOL tablet, film-coated, 2024). Bisoprolol levels may be decreased *via* CYP3A4 induction (Maideen et al., 2021). Moreover, these DDIs are theoretically implied, and until further evidence becomes available, *a priori* dose adjustments are not deemed necessary.

##### 3.2.2.4 Antiarrhythmics

Doravirine did not show any potential DDIs from either database. Nevirapine exhibited amber-class interactions with most antiarrhythmics (i.e., amiodarone, bepridil, digoxin, disopyramide, dronedarone, propafenone, and quinidine) when queried in the UoL database, likely due CYP3A4 induction, but no dose adjustments are recommended. Efavirenz exhibited the most important DDIs. The UoL database suggests avoiding amiodarone co-administration, whereas Lexidrug® suggests drug monitoring due to CYP3A4 induction lowering amiodarone concentrations (PK-DDI) and QT prolongation risk of both drugs (PD-DDI). Amiodarone prescribing information suggests that agents prolonging the QT interval should be avoided in co-administration with amiodarone (AMIODARONE HYDROCHLORIDE tablet, 2021). For patients at risk for arrhythmias or already receiving other QT-prolonging medications, caution and close monitoring are advised. Given that a multitude of agents may prolong the QT interval, the simpler strategy would probably be to discontinue efavirenz. QT prolongation is also the underlying amber-class PD-DDI of efavirenz with bepridil, disopyramide, dronedarone, flecainide, propafenone, and quinidine, according to the UoL database. In contrast, Lexidrug® reports these interactions as either yellow (treatment should be monitored) or blue (no action needed). However, due to the severity of a potential episode of torsades de pointes and the likelihood of polypharmacy, especially in elderly PLWH (Ruellan et al., 2021), care should be taken when administering efavirenz with these medications.

Etravirine may reduce the plasma concentrations of most antiarrhythmics due to CYP3A4 induction, but it can increase the concentration of digoxin through P-gp inhibition (INTELENCE—etravirine tablet, 2023). Prescribing information recommends the lowest possible dose when initiating digoxin while on etravirine. If digoxin is already part of the patient’s regimen, no dose adjustments are recommended; instead, only serum digoxin concentration monitoring and concomitant dose titration are recommended. Rilpivirine can also prolong QTc (in supratherapeutic doses), and the prescribing information warns against (or advises caution) co-administration with other QTc-prolonging factors (EDURANT—rilpivirine hydrochloride tablet, film-coated, 2024b).

##### 3.2.2.5 Antiplatelets and anticoagulants

Doravirine and rilpivirine showed no potential DDIs. Nevirapine showed potential interactions only in the UoL database with clopidogrel and ticagrelor. Clopidogrel transforms into its active metabolite through CYP2C19, whereas several other CYPs (e.g., 1A2, 2B6, 2C9, and 3A4) contribute to its metabolism to a lesser extent. CYP34 can be induced by nevirapine; therefore, altering clopidogrel’s metabolism and its active metabolite while inhibiting CYP2B6 by clopidogrel could increase nevirapine, but further studies are needed (Egan et al., 2014; PLAVIX—clopidogrel tablet, film-coated, 2022; Richter et al., 2004; Zhou et al., 2015). Ticagrelor levels may increase *via* a similar mechanism. Efavirenz and etravirine appear to interact similarly with clopidogrel and, by extension, with ticlopidine and ticagrelor. Induction of glucuronidation by efavirenz and etravirine could potentially reduce the levels of dipyridamole (DIPYRIDAMOLE tablet, 2023; Lee et al., 2012).

As for anticoagulants, rilpivirine and etravirine P-gp inhibition may increase dabigatran levels (INTELENCE—etravirine tablet, 2023; Moss et al., 2013; Weiss and Haefeli, 2013). Although CYP3A4 induction by efavirenz, etravirine, and nevirapine may reduce the concentrations of rivaroxaban and apixaban, the importance of the interaction varies between the two databases. Acenocoumarol and warfarin exhibit complex interactions with efavirenz, etravirine, or nevirapine *via* both CYP3A4 induction and CYP2C9 inhibition or induction by the abovementioned NNRTIs, which have competing effects on the “victim” drug concentration (EFAVIRENZ capsule, 2023; INTELENCE—etravirine tablet, 2023; VIRAMUNE—nevirapine tablet, 2022). For warfarin and the active S-enantiomer that is metabolized from CYP2C9, the extent of inhibition of CYP2C9 metabolism by NNRTIs should be considered. Close INR monitoring is required, and INR instability continues; switching the antiretroviral regimen, possibly to rilpivirine or doravirine, may be a prudent option when an NNRTI is required.

##### 3.2.2.6 Diuretics

Most diuretics exhibited a low DDI risk. Doravirine and rilpivirine presented no potential DDIs with any diuretic. Lexidrug® does not identify DDIs between NNRTIs and diuretics. In the UoL database, a potential PD-DDI between hydrochlorothiazide and efavirenz is reported, which is related to QT prolongation (EFAVIRENZ capsule, 2023; Seyerle et al., 2018). Eplerenone exhibited potential PK interactions due to NNRTI-mediated CYP3A4 induction with efavirenz, etravirine, and nevirapine (“amber” class).

#### 3.2.3 Integrase nuclear strand transfer inhibitors

**FIGURE 4:**
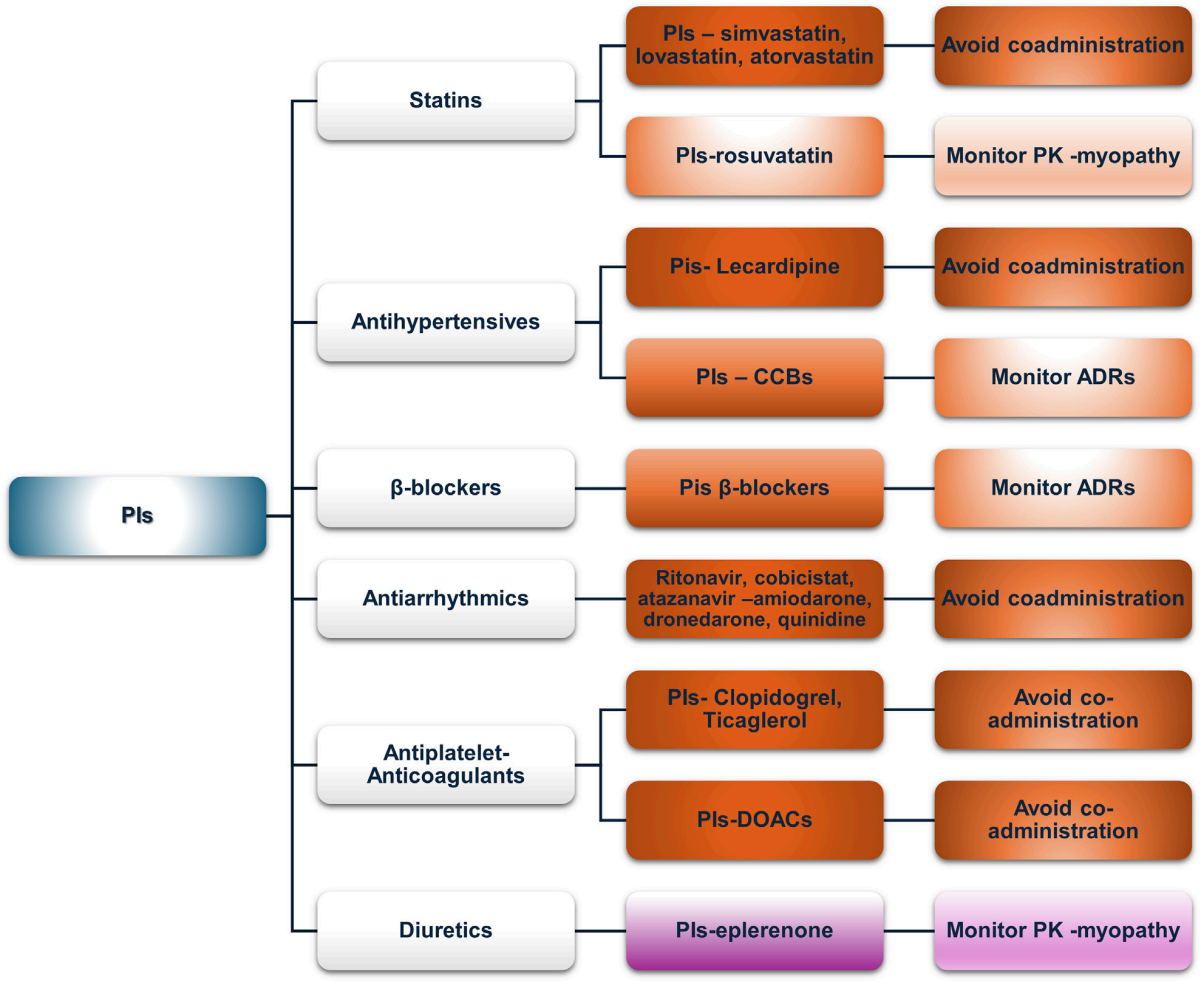
An overview of the potential DDIs between INSTIs and CVD medications with the most prominent cases and the suggested action according to the checkers used and literature data (green, no DDIs; light purple, potential but of low significance; red, clinically significant; dark red, avoid).

Integrase nuclear strand transfer inhibitors (INSTIs), apart from being a first-line treatment of HIV infection according to European and American guidelines, exhibit a favorable interaction profile, as evidenced by the analysis and corroborated by literature sources and expert opinions (Figure 4) (Askari et al., 2023; Wang, 2022). However, clinically significant DDIs can still occur, especially in older PLWH or people who experience polypharmacy. A cross-sectional study in Taiwan, consisting of more than a thousand PLWH and using the UoL database, found that over 10% of patients receiving INSTI regimens had red or amber DDIs, with those at higher risk being the elderly or patients with polypharmacy (Peng et al., 2024). These findings align with other studies showing a higher prevalence of potential DDIs, especially in people receiving multiple medications or those with comorbidities, even when using an INSTI-based regimen (Altunal et al., 2023; Kunimoto et al., 2021; Tinggaard et al., 2023). INSTIs are substrates of UGT and CYP3A enzymes; therefore, co-administration with strong inhibitors or inducers should be closely monitored if not completely avoided (Di Perri et al., 2019; Lu et al., 2021). Furthermore, dolutegravir and bictegravir inhibit MATE1 and OCT2, with dolutegravir also being a substrate of P-gp and BCRP. Therefore, plasma concentrations of medications eliminated *via* the OCT2 or MATE1 pathways can be increased when co-administered with these agents (BIKTARVY—bictegravir sodium, emtricitabine, and tenofovir alafenamide fumarate tablet, 2024; TIVICAY—dolutegravir sodium tablet, film-coated, 2024; Di Perri et al., 2019).

##### 3.2.3.1 Statins

No PK-DDIs were found in our analysis. Lexidrug® identified a PD-DDI with raltegravir as a potential risk for myopathy, which is associated with all statins. Reports of elevated CPK or myopathy with raltegravir are included in prescribing information (Calza et al., 2014; Cirioni et al., 2013; Lee et al., 2013; Madeddu et al., 2015; Monaghan et al., 2021; Tsai et al., 2016). Careful monitoring for symptoms of myopathy, such as fatigue, muscle pain, tenderness or weakness, nocturnal cramping, and tendon pain, is advised for PLWH receiving raltegravir and statins. Elvitegravir, when boosted with cobicistat, may lead to increased pitavastatin concentrations, but this is due to cobicistat’s inhibition of OATP1B3 (LIVALO—pitavastatin calcium tablet, film-coated, 2024b; TYBOST-cobicistat tablet, film-coated,” 2021). However, the net effect is unknown since elvitegravir has opposite effects with cobicistat (GENVOYA—elvitegravir, cobicistat, emtricitabine, and tenofovir alafenamide tablet, 2022).

##### 3.2.3.2 Antihypertensives (ACE inhibitors, ARBs, and CCBs)

The Lexidrug® database shows no potential DDIs, but the UoL database showed possible DDIs with bictegravir as a victim drug due to verapamil- or diltiazem-mediated P-gp and CYP3A4 inhibition (low clinical relevance). Bictegravir is available as a co-formulation with tenofovir alafenamide (Huliciak et al., 2023), and both have a favorable toxicity profile in a wide range of concentrations, even supratherapeutic (Gallant J. et al., 2017; Gallant J. E. et al., 2017; Sax et al., 2017). Elvitegravir, as a moderate inducer of CYP2C9, may decrease azilsartan, irbesartan, or losartan concentrations according to the UoL database but not according to Lexidrug® (STRIBILD-elvitegravir, cobicistat, emtricitabine, and tenofovir disoproxil fumarate tablet, film-coated, 2021).

##### 3.2.3.3 β-blockers

Bictegravir inhibits OCT2 and dolutegravir inhibits MATE1, which could potentially modulate the active renal excretion of atenolol or sotalol, as pointed out by the UoL database. These data have been suggested as a similar mechanism as in the case of metformin, with various percentages of increase depending on either dolutegravir or bictegravir. Dose adjustments may be required but are not mandatory (BIKTARVY- bictegravir sodium, emtricitabine, and tenofovir alafenamide fumarate tablet, 2024; TIVICAY- dolutegravir sodium tablet, film-coated, 2024; Di Perri et al., 2019).

##### 3.2.3.4 Antiarrhythmics

The Lexidrug® database did not show any potential DDIs. The UoL database flagged DDIs between disopyramide with bictegravir or dolutegravir (mediated by OCT2 or MATE1 inhibition, see above) and bictegravir with quinidine (P-gp inhibitor), but all of which are of low clinical significance. Disopyramide is partially metabolized by CYP3A4, so no *a priori* dose adjustment is needed except limited caution, whereas bictegravir has a broad therapeutic window. Furthermore, co-formulated administration of bictegravir with TAF does not require any dose adjustments (Gallant J. et al., 2017; Gallant J. E. et al., 2017; Huliciak et al., 2023; Sax et al., 2017).

##### 3.2.3.5 Antiplatelets and anticoagulants

Elvitegravir moderately induces CYP2C9, potentially reducing the concentrations of clopidogrel, acenocoumarol, or warfarin. However, as elvitegravir is available only in cobicistat-boosted regimens, DDIs with cobicistat should be considered *a priori*. Hence, elvitegravir formulations should not be administered with clopidogrel as they reduce the AUC of clopidogrel’s active metabolite and impair platelet aggregation. Clinical data (with boosted PIs) also suggest a loss of clopidogrel’s efficacy with these regimens (see the PI section).

##### 3.2.3.6 Diuretics

Amiloride excreted *via* OCT2 transporters could interact with dolutegravir or bictegravir, but according to the UoL database, this potential DDI is clinically insignificant (BIKTARVY—bictegravir sodium, emtricitabine, and tenofovir alafenamide fumarate tablet, 2024; TIVICAY—dolutegravir sodium tablet, film-coated, 2024; Di Perri et al., 2019).

#### 3.2.4 Protease inhibitors

**FIGURE 5:**
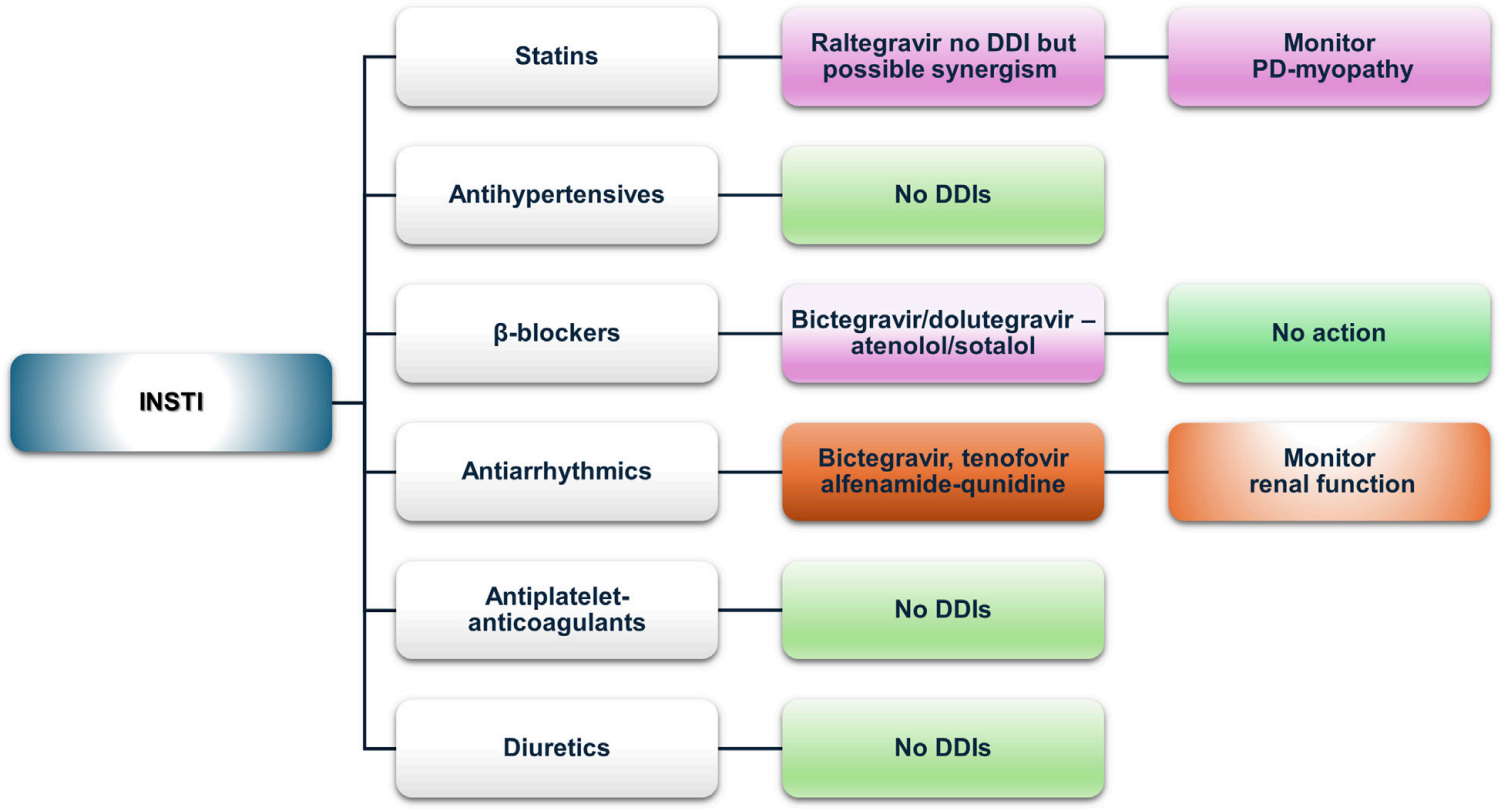
An overview of the potential DDIs between PIs and CVD medications with the most prominent cases and the suggested action according to the checkers used and literature data (green, no DDIs; light purple, potential but of low significance; red, clinically significant; dark red, avoid).

Although integrase inhibitors dominate evidence-based prescription practices for PLWH, protease inhibitors (PIs) remain widely used, especially in older populations. They act directly on the HIV protease, and ever since their advent in 1995, HIV-associated mortality has been reduced dramatically (Palella et al., 1998). Their inhibiting properties in various proteins and transporters, i.e., P-gp, and the strong inhibitory effects of “booster” co-drugs (ritonavir and cobicistat) on CYPs (i.e., CYP3A4 and CYP2D6) give rise to a multitude of potential PK-DDIs, often complicating co-prescription with other drug classes (Tamemoto et al., 2023). As for their implication in potential PD-DDIs, PIs prolong the QTc interval, especially when co-administered with other QT-prolonging medications, likely due to hERG channel blockage, as has been shown *in vitro* (Anson et al., 2005). Figure 5 presents an overview of the clinically significant DDIs that are discussed further below.

##### 3.2.4.1 Statins

Our analysis found concordance in the absolute contraindication of co-administering lovastatin and simvastatin with any PI. Most other PI–statin combinations were marked as “amber” or “yellow,” consistent with the prescribing information of most PIs. Atorvastatin is usually contraindicated, but if deemed necessary, it may be used in limited doses, such as 10 mg max with cobicistat formulations or 20 mg with unboosted darunavir/atazanavir formulation (GENVOYA—elvitegravir, cobicistat, emtricitabine, and tenofovir alafenamide tablet, 2022; KALETRA—lopinavir and ritonavir tablet, film-coated, 2023). Rosuvastatin, although poorly metabolized by CYP3A, shows increased AUC and C_max_ when co-administered with lopinavir/ritonavir, which is likely due to the inhibition of transporters mediating rosuvastatin distribution, necessitating dose adjustments or switching to other statins (Elsby et al., 2023; KALETRA—lopinavir and ritonavir tablet, film-coated, 2023; TYBOST—cobicistat tablet, film-coated, 2021). Fluvastatin and pravastatin, with minimal CYP-mediated metabolism, appear to have a better profile among the statin group with lopinavir/ritonavir, recommending these medications in case statin therapy is indicated (KALETRA—lopinavir and ritonavir tablet, film-coated, 2023). However, in cobicistat co-formulated PIs (atazanavir/cobicistat, darunavir/cobicistat), a potential DDI of “amber” or “yellow” significance is reported due to OATP1B1 inhibition, but atazanavir prescribing information still suggests these statins as a safer alternative (REYATAZ—atazanavir capsule, gelatin-coated, 2023). Pitavastatin is minimally metabolized by CYPs, but it is subject to glucuronidation. Thus, reduced concentrations of pitavastatin may be observed due to the induction of glucuronidation from lopinavir/ritonavir formulations or increased concentrations when atazanavir is co-administered. These predicted changes are not clinically significant, and they are shown similarly in Lexidrug®, whereas the UoL database does not report them.

##### 3.2.4.2 Antihypertensives (ACE inhibitors, ARBs, and CCBs)

ACE inhibitors (ACEIs) and ARBs appear to exhibit the most favorable interaction profile, compared to CCBs, which are more affected by PI-mediated CYP3A4 inhibition. Lercanidipine is contraindicated with any strong CYP3A4 inhibitor, and that is reflected in the product SmPC (Zanidip (Lercanidipine hydrochloride) tablets—Summary of Product Characteristics, 2022). Other CCBs (e.g., amlodipine, lacidipine, nifedipine, verapamil, and diltiazem) require close clinical monitoring, as suggested by both databases, which is consistent with the SmPCs of the PIs analyzed, due to the risk for augmented hypotension and probable additive effect to PR and QT prolongation (KALETRA—lopinavir and ritonavir tablet, film-coated, 2023; NORVIR—ritonavir tablet, film-coated, 2022; PREZISTA-darunavir tablet, film-coated, 2023; REYATAZ—atazanavir capsule, gelatin-coated, 2023). In some cases, i.e., atazanavir, a dose titration and ECG monitoring should be followed (REYATAZ—atazanavir capsule, gelatin-coated, 2023). Among ACEIs, potential DDIs are sparse and reported only *via* the UoL database. Benazepril, a UGT substrate, may be affected by atazanavir’s inhibition of UGT1A1 (REYATAZ—atazanavir capsule, gelatin-coated, 2023). Valsartan, an OATP1B1 and MRP2 substrate, may also be affected by the inhibitory effect of PIs (DIOVAN—valsartan tablet, 2023; Elsby et al., 2023). Regarding ARBs, irbesartan, losartan, and azilsartan concentrations may be lowered due to weak induction of CYP2C9 from ritonavir, but the clinical significance remains unclear, whereas the DDI is reported only in the UoL database (COZAAR—losartan potassium tablet, film-coated, 2022; EDARBI—azilsartan kamedoxomil tablet, 2024a).

##### 3.2.4.3 β-blockers

Potential DDIs were mostly retrieved from the UoL database. All analyzed β-blockers exhibited PK or PD-DDIs with atazanavir, and most showed interactions with other PIs. PK-DDIs involved CYP3A4 or CYP2D6 inhibition (increased exposure), transporters, or UGT enzymes. PD-DDIs were related to risk for QT prolongation, particularly with atazanavir or lopinavir. Atazanavir does not interact with CYP2D6 substrates such as metoprolol, carvedilol, or nebivolol, and only PD interactions are expected (REYATAZ-atazanavir capsule, gelatin-coated, 2023). The UoL database classifies the interactions between β-blockers and either atazanavir or lopinavir as “amber,” whereas Lexidrug® showed no clinically significant DDIs, which is in line with atazanavir’s SmPC that reports no expected clinically significant effect for atenolol.

##### 3.2.4.4 Antiarrhythmics

Antiarrhythmics potentially exhibit a multitude of clinically significant DDIs, with many combinations characterized as contraindicated when ritonavir, cobicistat, and atazanavir are co-prescribed. Amiodarone, dronedarone, and quinidine have the most absolute contraindications, primarily due to the inhibition of CYP3A4 or CYP2D6. In addition, due to the possible effect of PIs on the QT (especially ritonavir or atazanavir), ΕCG monitoring is essential. For digoxin, P-gp inhibition by cobicistat-boosted regimens may result in increased exposure, mandating close monitoring of digoxin plasma levels. As for ritonavir-boosted regimens, ritonavir has a biphasic effect, with initial inhibition being followed by the induction of P-gp. Therefore, if ritonavir regimens are followed by digoxin initiation, the therapeutic effect may be delayed (NORVIR-ritonavir tablet, film-coated, 2022; TYBOST-cobicistat tablet, film-coated, 2021).

##### 3.2.4.5 Antiplatelets and anticoagulants

Ticagrelor should not be co-administered with PIs due to CYP3A4 inhibition. The UoL database revealed an absolute contraindication between clopidogrel and either ritonavir- or cobicistat-boosted regimens, mediated by CYP3A4 inhibition; however, Lexidrug® only shows it as “amber,” possibly because the main metabolic activation of clopidogrel is through CYP2C9. The darunavir prescribing information suggests that it is “not recommended.” Prasugrel undergoes similar metabolism but does not interact significantly, requiring no dose adjustments (PREZISTA-darunavir tablet, film-coated,” 2023). Aspirin and dipyridamole do not exhibit any potential DDIs.

DOACs exhibit a multitude of interacting mechanisms, which is also reflected in our analysis (Tamemoto et al., 2023). All DOACs are expected to interact with cobicistat (Gordon et al., 2016). Rivaroxaban should be avoided with cobicistat- or ritonavir-boosted regimens due to increased exposure from CYP3A4 and P-gp inhibition (KALETRA-lopinavir and ritonavir tablet, film-coated, 2023; NORVIR-ritonavir tablet, film-coated, 2022; TYBOST-cobicistat tablet, film-coated, 2021; Yoong et al., 2017). Nevertheless, a PK analysis found that rivaroxaban can be continued in patients receiving short-term ritonavir (Rohr et al., 2024). Additionally, considering a CYP3A4 recovery half-time of approximately 2–3 days may help guide medication reintroduction (Rohr et al., 2024). For example, in the case of nirmatrelvir/ritonavir, a gradual reinstitution of atorvastatin within 4 days from discontinuing nirmatrelvir/ritonavir was deemed safe (Krohmer et al., 2023). Apixaban may require a 50% dose reduction when co-administered with strong CYP3A4 inhibitors (ELIQUIS- apixaban tablet, film-coated, 2021). Dabigatran and edoxaban, as P-gp substrates, may exhibit increased exposure when co-administered with cobicistat (Gordon et al., 2016). Our analysis shows different DDI designations for dabigatran and ritonavir (“amber” according to the UoL and “unknown” according to the Lexidrug® databases). A population-based analysis suggested that dabigatran should not be given to adults with severe renal impairment receiving ritonavir or cobicistat, whereas a dose reduction to 110 mg twice daily or 2-h separation from ritonavir dosing and a further dose reduction to 75 mg twice daily in the case of cobicistat co-administration are recommended for patients with moderate renal impairment. For normal renal function, no dose reduction is necessary with ritonavir, but for cobicistat, a dose reduction to 110 mg twice daily is suggested (Lingineni et al., 2021). In the case of warfarin, CYP2C9 induction led by ritonavir, which potentially decreases S-warfarin (the active enantiomer) concentration, and CYP3A4 inhibition, which can increase warfarin concentration, are the main mechanisms that drive the potential DDIs. However, the effects and clinical significance of this potential interaction are unclear. A case report presented data of sub-therapeutic INR levels following the initiation of elvitegravir/cobicistat, showing that in this case, elvitegravir-associated CYP2C9 induction was the leading cause behind the observed effect, rather than cobicistat-associated CYP3A4 inhibition, showing that “potent inhibition” does not always mean that it will prevail in mixed drug interactions such as this, but further data are needed (Good et al., 2015). In the case of existing clinical data concerning cobicistat, the effect was that of prolonged INR (Tseng et al., 2017). Given that common practice dictates frequent monitoring of INR, a more vigilant and maybe more frequent INR measurement when initiating or discontinuing warfarin is prudent. Acenocoumarol shows no significant DDIs, nor is it mentioned in PIs’ SmPC discussed, although it is mainly metabolized through CYP2C9. The UoL database attributes an “amber” designation for the potential interaction, based on the PK profile and a case report (Llibre et al., 2002). However, a literature search retrieved only two other case reports, one of which was in an HCV-treated patient and the other was initially treated with efavirenz (De Lorenzo-Pinto et al., 2016; Welzen et al., 2011). Given the existence of such reports, and until further evidence is available, caution is advised for patients receiving ritonavir-boosted regimens and acenocoumarol.

##### 3.2.4.6 Diuretics

Eplerenone and protease inhibitor co-administration is contraindicated due to CYP3A4 metabolism. In a case report by Cordova et al. (2021) highlighting the importance of this DDI, severe hyperkalemia occurred in a PLWH receiving eplerenone with ritonavir, which subsided when ART was switched to a dolutegravir-containing regimen.

#### 3.2.5 Attachment–post attachment inhibitors and entry inhibitors

**FIGURE 6:**
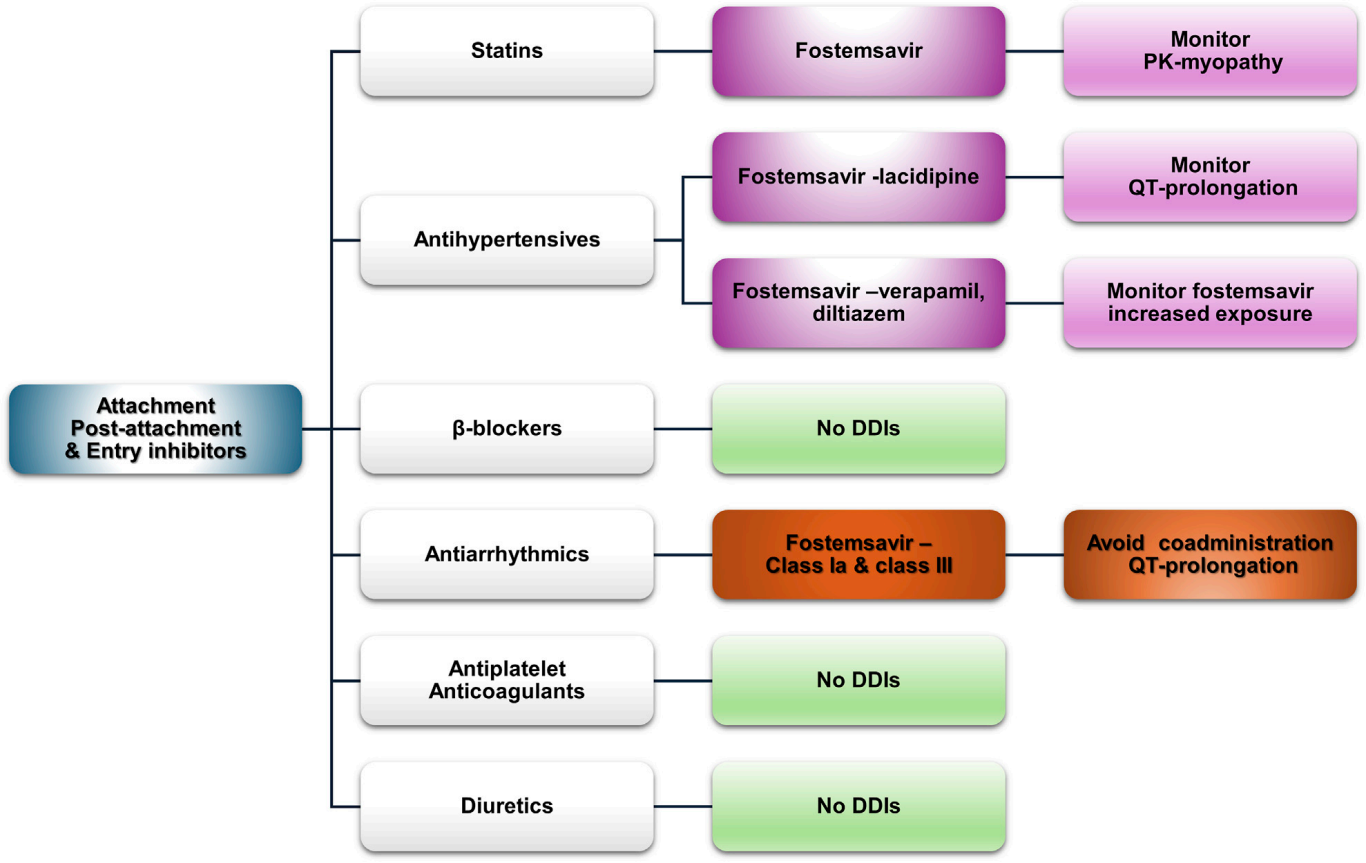
An overview of the potential DDIs between attachment-post attachment inhibitors and entry inhibitors and CVD medications with the most prominent cases and the suggested action according to the checkers used and literature data (green, no DDIs; light purple, potential but of low significance; red, clinically significant; dark red, avoid).

Fostemsavir is a first-in-class, gp-120-directed attachment inhibitor for heavily treatment-experienced PLWH with multidrug-resistant HIV. It is metabolized by CYP3A4 to its active form, temsavir. Co-administration with inhibitors or inducers is expected to alter temsavir plasma concentrations (RUKOBIA-fostemsavir tromethamine tablet, film-coated, extended release, 2024). Maraviroc is a CCR5 co-receptor antagonist indicated for CCR5 tropic HIV1 that is also metabolized by CYP3A, and it requires dose adjustments upon co-administration with CYP3A inducers or inhibitors (SELZENTRY- maraviroc tablet, film-coated, 2022). In initial studies with maraviroc in a treatment-experienced population, coronary heart disease occurred more often in the drug arm than in the placebo arm, while a longer (>5 years) analysis of the MOTIVATE trial population reported myocardial ischemia rates similar to those in the initial study (Gulick et al., 2014). Experimental evidence and clinical evidence suggest that maraviroc may favorably modulate atherosclerosis, but high costs for tropism testing and newer antiretrovirals limit its use (Cipriani et al., 2013; De Luca et al., 2019; Francisci et al., 2019; Piconi et al., 2016). Figure 6 summarizes the potential clinically significant DDIs of fostemsavir and maraviroc with CVD medications.

##### 3.2.5.1 Statins

Fostemsavir has “amber”-class DDIs with all statins in both databases, except pravastatin in the UoL database, as it is not a substrate of BCRP. OATP and BCRP inhibition may increase statin exposure and the risk for related side effects such as myopathy; thus, caution is advised. Maraviroc does not exhibit any potential side effects according to both databases.

##### 3.2.5.2 Antihypertensives

Fostemsavir may act additively with lacidipine in increasing QT. Verapamil and diltiazem inhibit CYP3A4 and can increase the concentration of both fostemsavir and maraviroc. For maraviroc, guidance exists regarding the appropriate dose reduction (SELZENTRY- maraviroc tablet, film-coated, 2022). The UoL database assigns an amber designation to olmesartan, valsartan, and telmisartan when co-administered with fostemsavir because of OAT1B1 inhibition, which may increase the concentrations of these ARBs.

##### 3.2.5.3 β-blockers

No DDIs are expected with sotalol, which is discussed alongside the other antiarrhythmics as a class III agent.

##### 3.2.5.4 Antiarrhythmics

Class-Ia and class-III antiarrhythmics (i.e., amiodarone, sotalol, disopyramide, and quinidine), along with flecainide and dronedarone, are potentially augmented by fostemsavir and should be avoided unless a risk–benefit analysis suggests otherwise. The Lexidrug® and UoL databases attribute different degrees of severity to these potential interactions. The SmPC of fostemsavir recommends close monitoring in the case of co-administration (RUKOBIA-fostemsavir tromethamine tablet, film-coated, extended release, 2024). However, to the best of our knowledge, no other data exist in the literature. Dronedarone, a potent inhibitor of P-gp (MULTAQ-dronedarone tablet, film-coated, 2023), can affect the metabolism of temsavir; however, as the fostemsavir SmPC states, it can be co-administered with P-gp and CYP3A inhibitors (RUKOBIA-fostemsavir tromethamine tablet, film-coated, extended release, 2024). It can also increase maraviroc exposure through moderate inhibition of CYP3A4.

##### 3.2.5.5 Antiplatelets and anticoagulants

No potential DDIs are expected with antiplatelet therapies. As for anticoagulants, apixaban and rivaroxaban are substrates of BCRP, inhibition of which (from fostemsavir) may lead to increased exposure to these DOACs (RUKOBIA-fostemsavir tromethamine tablet, film-coated, extended release, 2024).

##### 3.2.5.6 Diuretics

No potential DDIs are expected.

## 4 Discussion

Advancements in HIV treatment have led to longer life expectancies for PLWH, making age-related chronic conditions such as CVDs increasingly common. HIV pathophysiology itself predisposes patients to CVD, with mechanisms including HIV-associated dyslipidemia, endoplasmic reticulum stress, inflammasome activation, and autophagy dysregulation (Kearns et al., 2017). The virus directly affects blood vessels and disrupts the function of macrophages and endothelial cells (Gibellini et al., 2013). HIV-induced inflammation, immune activation, and oxidative stress further contribute to atherogenesis and cardiovascular impairment, amplifying the overall CVD risk for PLWH compared to that of the general population (Kearns et al., 2017; Nou et al., 2016; Triant, 2013). In addition, traditional CVD risk factors, such as lifestyle choices, exacerbate CVD risk in this population. In daily clinical practice, such overlapping risks demand personalized assessment and close monitoring when initiating or adjusting therapy. Co-administration of ART and CVD medications is often necessary, making the management of DDIs crucial for minimizing ADRs, and comprehensive guidance on managing therapeutic protocols for PLWH with CVD or other comorbidities is increasingly being discussed (Amariles et al., 2023; Giguère et al., 2019). Clinicians should integrate pharmacological data with patient-specific factors, comorbidities, and treatment goals to optimize safety. Flexibility in ART regimen adjustments is essential as HIV treatment often takes precedence. Physicians must also tailor the treatment of comorbidities such as CVD and adjust treatment accordingly to ensure optimal therapeutic outcomes. In this review, we analyze and discuss DDIs between ART and CVD medications, utilizing two widely used databases that are often used by healthcare professionals as tools for evidence-based treatment decision-making in HIV treatment. Although DDI checkers offer useful insights, integrating this information into a patient-specific clinical decision-making process remains a key challenge. Translating DDI checker outputs into actionable clinical decisions requires contextual judgment, particularly in complex or polymedicated patients.

In clinical practice, real-life implications of combining CVD risk-modifying medications with antiretrovirals may lead to adverse outcomes either through direct toxicity or functional impairment (Ambrosioni et al., 2024). Proactive monitoring and vigilance for potential DDIs between ART and other medications and antiretrovirals is essential for avoiding HIV treatment discrepancies or toxicity from co-administered ART, CVD, or other medications. Treating physicians often consult DDI checkers. In clinical practice, what is needed is the final clinical information or, in other words, how the information presented by available DDI checkers, even with a user-friendly format, can be incorporated into a clinical decision-making process. This is particularly important when inconsistencies between databases require further analysis and the integration of additional elements (Conti et al., 2024; Helmons et al., 2015; Shareef et al., 2021).

The use of two complementary resources allowed us to maintain analytical depth, clarity, and focus, balancing specialized HIV pharmacology coverage (UoL) with a broader clinical pharmacotherapy scope (Lexidrug®). Including additional databases, although feasible, might have overcomplicated the comparative analysis and shifted the focus of the current work toward a more quantitative database-comparison approach rather than preserving its qualitative and interpretive emphasis on clinical relevance. Our analysis revealed that the two databases were not always in full concordance, particularly regarding the clinical significance of certain DDIs (Figure 1). This discrepancy is consistent with other studies comparing DDI databases and may stem from variations in data reporting or analysis (Drwiega et al., 2022; Ramos et al., 2015; Suriyapakorn et al., 2019; Vivithanaporn et al., 2021). While databases had a relatively high concordance concerning the absolute contraindications, the UoL database highlighted more DDIs, particularly for statins, antiarrhythmics, and anticoagulants, though the level of evidence was often very low. SmPCs of older drugs, including antiretrovirals, often lacked an exhaustive list of potential DDIs. Both checkers provide useful information for treating physicians, but a solid understanding of pharmacokinetics, drug metabolism, and therapeutic drug monitoring is needed, along with vigilance to ensure that patients receive optimum treatment.

Proactively evaluating DDIs in PLWH is a crucial aspect and should not be underestimated by healthcare providers. With the increasing focus on CVD risk management in PLWH, clinically significant interactions between ART and CVD medications require active monitoring. Results from a recent cohort study report that ART–CVD DDIs were the third most prevalent, after systemic anti-infectives and central nervous system medications. Caution is advised with interactions including clopidogrel, simvastatin, amiodarone, and apixaban, especially when co-administered with boosted elvitegravir, atazanavir, and PIs (De Oliveira Costa et al., 2023). The Swiss HIV cohort and other case studies have also identified CVD medications as the most commonly involved drugs in DDIs, especially with boosted ART regimens, leading to potential risks (Al Sayed et al., 2022; Deutschmann et al., 2021; El Moussaoui et al., 2020; Okoli et al., 2020). Apart from boosted ART regimens, additional risk factors include age, polypharmacy, and underlying comorbidities (Al Sayed et al., 2022; El Moussaoui et al., 2020).

NRTIs continue to be integral to HIV treatment guidelines due to their effectiveness and relatively low interaction potential, which highlights their clinical utility in diverse patient populations (DESCOVY—emtricitabine and tenofovir alafenamide tablet, 2022; EACS Guidelines|EACSociety, 2024; VIREAD ACCESS—tenofovir disoproxil fumarate tablet, coated, 2021). They exhibit no significant DDIs with commonly prescribed medications such as statins or antihypertensives, though caution is advised with verapamil, which may increase tenofovir exposure. Minor discrepancies between the databases highlight the importance of clinical evaluation of patient characteristics (Drwiega et al., 2022; Ramos et al., 2015; Ruellan et al., 2021; Suriyapakorn et al., 2019; Vivithanaporn et al., 2021). NNRTIs, primarily metabolized by CYP enzymes, present varying interaction potentials. Doravirine and rilpivirine exhibit minimal DDIs, while efavirenz, etravirine, and nevirapine require close monitoring, especially with statins, antihypertensives, and antiarrhythmics. Efavirenz has notable DDIs with simvastatin and amiodarone, requiring careful monitoring. Overall, doravirine and rilpivirine appear to be safer options in terms of DDIs. Other NNRTIs require closer management to mitigate risks (Egan et al., 2014; Gerber et al., 2005; Hosseini et al., 2021; Kakuda et al., 2011; Lee et al., 2012). INSTI-based regimens seem to be the safest in terms of DDIs. INSTIs are metabolized by UGT and CYP3A enzymes and can be affected if co-administered with strong inhibitors or inducers, particularly in older patients or those on multiple medications (Gallant J. et al., 2017; Gallant J. E. et al., 2017; Sax et al., 2017). Dolutegravir and bictegravir also inhibit MATE1 and OCT2, affecting drugs eliminated *via* these pathways. INSTIs can be generally administered with statins, antihypertensives, and anticoagulants, but close monitoring is recommended, especially for raltegravir due to its myopathy risk. PIs, particularly those with boosting agents, have the highest potential for clinically significant DDIs and require additional caution when prescribing PIs to patients with or at risk for CVD (Carr et al., 1999; Carr and Cooper, 2000; Grinspoon and Carr, 2005; Stein et al., 2001). Statins and antiarrhythmics exhibited the most potential DDIs, with lovastatin, simvastatin, and amiodarone having the most absolute contraindications. PD-DDIs, such as QT prolongation, and PK-DDIs increasing the risk of adverse effects from medication, such as statin-associated myopathy, hepatotoxicity, or increased INR, are the most common potential associated adverse events. Attachment and entry inhibitors, such as fostemsavir and maraviroc, which are used in multidrug-resistant HIV or for specific CCR5 tropism, can be the victim drugs in cases of co-administration with CYP3A4 inhibitors or inducers (De Luca et al., 2019; Kozal et al., 2020). Fostemsavir may also elevate QT prolongation risk and partially interact with anticoagulants such as apixaban and rivaroxaban through the inhibition of BCRP, but no significant DDIs are expected with antiplatelets or diuretics.

Strengthening awareness of ART–CVD DDIs can support evidence-based prescribing and reduce preventable adverse drug events in HIV care. The strength of this review lies in its critical examination of the interactions between ART and a range of commonly used medications for CVD rather than focusing solely on one class of ART or CVD drugs. It also adds value by discussing first-in-class drugs such as fostemsavir, cabotegravir, and rilpivirine and exploring cutting-edge treatments in this field. Our approach incorporates data from two widely used DDI checkers, combined with available literature data, to provide a more critical of the clinical significance of potential DDIs. Nevertheless, some limitations should be acknowledged, such as the exclusion of other drug databases and the arbitrary nature of the included list based on clinical experience, which may have introduced bias into the discussion. This work also does not discuss potential DDIs within ART regimens or among CVD medications, which, although potentially crucial in clinical practice, were deemed outside the scope of this work. Despite these, the review offers valuable insights into ART–CVD medication interactions, making it a useful resource for healthcare professionals.

## 5 Conclusion

Our work discusses the complex landscape of DDIs between ART therapies and CVD medications. While antiretrovirals such as INSTIs and NRTIs offer safer options with fewer interaction risks, PIs and some NNRTIs still require careful management. Monitoring and dose adjustments are essential to avoid adverse outcomes and ensure optimal treatment for PLWH, especially those with comorbid CVD. Clinicians must remain vigilant in incorporating DDI data from multiple sources into their decision-making processes, thus combining pharmacological knowledge with clinical assessment to provide safe and effective care.
